# A User-Centred Design Approach to Integrated Information Systems – A Perspective

**DOI:** 10.5334/ijic.4182

**Published:** 2018-05-22

**Authors:** Niamh Lennox-Chhugani

**Affiliations:** 1 Integrated health and care lead, Optimity Advisors, EMEA, 4-6 Nile St, London, GB

**Keywords:** Integrated care, Integrated information, user-centred design, user needs

## Abstract

Integrated care requires joined up information for effective planning, delivery and management. Different countries are experimenting with a range policy approaches to designing integrated information systems with varying levels of success. Information systems to support integrated care that start with the needs of the user of information (individual, health and care professional, service manager, payer, policy maker), are most likely to succeed in being adopted. We suggest that using the MINDSPACE behavioural framework alongside technology tools to engage users in a process of co-design for integrated information systems will help deliver on the promise of better population health and better value healthcare sooner rather than later by producing information systems that support real time coordinated person- and community-centred decision-making.

## Introduction

It feels very much like we are now at a tipping point for integrated care. No longer do those of us who have been advocating integration for decades need to convince policy-makers and senior decision-makers that taking a whole systems approach is the best way to plan and deliver health and care. In 2016, all 192 participating states endorsed the WHO Framework on integrated people-centred health services [1]. The recent *State of Health in the EU Companion Report* [2] pointed out that all EU countries are implementing integrated care albeit at different levels of scale and developing a wide variety of models. New models of integrated care are well established in the US, Singapore, Australia and New Zealand supported by changes to policy that create some of the conditions to enable integration.

## Integrated information systems

So how do we maximise the opportunity we have to scale integration and accelerate progress? I have been working as a clinician, service manager, advisor and academic in the field of integrated care for nearly 30 years and am acutely aware of the complexity of the challenge in scaling up integrated care. One area stands out above others as a particular challenge based on work in over a dozen health and care systems in England and our recent review of in excess of 600 integrated care initiatives across the EU [2]. Integrated information drives decision-making and care management, at community and at individual care user levels. As Ovretveit [3] put it information systems tend “to reinforce old models of care rather than “nudge” providers towards more beneficiary-centered and multidisciplinary working models”. It is therefore not surprising that anyone working in integrated care today will tell you that integrated information feels like a very distant ambition yet. Integrated information is necessary to share care planning, delivery and decision-making with health and care service users. Without it, coordinating care for service users across multiple providers is cumbersome and slow. It is essential to plan services around new and emerging needs of a community or population. Payers, be they government or insurers, need integrated information to understand if providers are meeting the needs and expectations of the community or population they are being paid to serve.

We have a good understanding of the challenges – multiple inherited IT systems that don’t talk to one another, information governance that gets in the way of sharing information between different providers of care, slow investment in mobile technologies to support care closer to home and weak understanding of how integrated data and information can support strategic planning for a reimagined health and care community. I have worked with one health system that, after spending two years convincing system leaders of the value of a single information management system to monitor performance, had to put into place 28 separate data sharing agreements, before this could happen.

We also know ***what*** needs to be done. There are examples of action in this space that show what can be achieved but delivering at scale remains rare [4]. I think this is because we don’t explicitly address the needs, desires and limitations of users of information for integration in health systems. We rarely (if ever) take a user-centred approach to designing information systems for integrated care.

## A user-centred design approach

Historically, systems that have sought to address needs and desires for integrated information adopt top-down, “carrot and stick” type approaches. There are some systems where there are explicit incentives. At one end of the population scale, in the US, the American Recovery and Reinvestment Act of 2009 [5] incentivised healthcare providers to use technology to coordinate care. From 2015, this switched to financial penalties for not being able to demonstrate meaningful use. In Iceland, the Health Records Act [6] set out the legislative requirement for all healthcare providers to use Electronic Health Records and share data through HealthNet in order to receive funding from the Directorate of Health. In Australia, using a similar approach to the US, they recently introduced an eHealth incentive [7] under their Practice Incentive Program for primary care providers. In England, incentives are agreed locally between Clinical Commissioning Groups (CCGs) and provider trusts often through the mechanisms of Commissioning for Quality and Innovation (CQUIN) payment conditional on meeting agreed targets. One current example related to integrated information is the incentivising of providers to publish and make available for referral all out-patient services on the NHS e-Referral service in order that GPs can use this route for referrals [8]. There are also implicit incentives provided in many systems that comprise of short term transformation funds to support the development of integrated information and whilst this can be a very powerful carrot to kick start initiatives, it is not sustainable as an incentive model.

Whilst these top-down approaches can set clear expectations for integrated information systems, they miss out an essential understanding of the needs and desires of the user of the information system that will ensure that data is collected accurately and on time, analysed meaningfully to inform real-time decision-making and the resulting outputs or feedback are an accepted shared version of the truth of integrated care practice.

In this paper, I have defined users at three levels, the integrated care service decision-maker (usually a service manager or commissioner/health insurer), the health or care provider (a front line member of the integrated care team) and the individual being provided with integrated care. Each of these will have different but fundamentally complementary needs and motivations. I have focused the discussion below on the first two levels, as this is where I have had most experience.

## A framework for understanding user needs and desires

The MINDSPACE framework [9] developed by the Behavioural Insights Unit at the Cabinet Office in collaboration with the Institute for Government in the UK is still a very helpful way to think about what drives behaviour in any context. The framework aims to understand how policy and programme implementation drives behaviour change using psychology, social psychology and economic theory. Applied as part of a user-centred design approach to integrated information systems, it helps with understanding the user (the persona), their journey through integrated care practice and it provides some useful insights into how we can think differently about data collection and management, analysis and sharing the resulting information to support decision-making to deliver integrated care. I have applied the framework when supporting integrated care systems on their developmental journey from early thinking about design to maturing models of integration at scale and use this experience to illustrate the framework components below.

**Social influence and norms:** Integrated care teams face conflicting pressures here at times. They want to share information with each other for the good of the person receiving care, but their separate employing organisations and professional regulation can require them to limit the information they can share. We have worked with integrated care teams that have two computers on their desks, one operating systems used by social care, the other health information systems with team members having to manually read across for each service user. Equally, integrated care managers and payers will use the information that they have always used, trust and understand to make decisions about priorities and action knowing that the regulators expect and inspect their performance using these information systems.

**Salience and priming:** Integrated care teams act on prompts in their immediate environment. If they have easy access to data collection tools, there is transparency around how data is managed, analysed and turned into information they need and can use, these create a virtuous circle of positive reinforcement. Conversely, systems today prompt them to work in organisational silo’s creating friction in the process of integrated working practices which I have observed teams designing manual work-arounds to overcome.

**Commitment and reciprocity:** Integrated care teams which have a shared sense of purpose find that this is reinforced when data and information about their performance in achieving this purpose is shared too. I have worked with integrated care teams in the early stages of designing performance dashboards that are meaningful to them in their and the individuals they serve. The first hurdle they have to overcome, is putting aside the requirements of the system for mandatory information which will be collected any way, and focusing solely on what they need to know to make the best decisions with and for individuals.

**Incentives and choice environment:** Integrated care teams are not incentivised to collect and analyse data in a transparent and integrated way that allows them to trust a shared version of the truth. This can be addressed through the process of collaborative design of integrated care team dashboards, providing teams with a shared version of the truth that enables them to make timely, person-centred, shared decisions. I have worked with teams who describe how even a manual version of such a design has transformed the way that they work together and with service users. I have had many conversations with provider and payer managers about the importance of accurate timely information to support the design of integrated care payment systems. I point out the if such as design is to become embedded and sustained, payers need to design short-term process incentives such as the meaningful use payments in the US, to ensure that those collecting the data understand why accurate, complete and up to date information is important to them, that is, it is directly linked to payments for integrated care.

**Figure 1:**
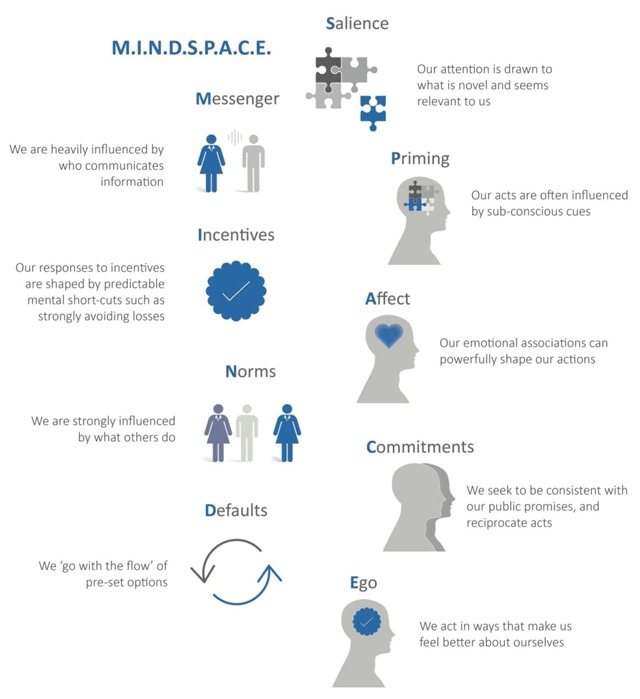
MINDSPACE influences.

The MINDSPACE framework suggests that there are 9 influences on behaviour and I have applied them when working with integrated care systems designing shared information dashboards and other management information frameworks supporting integrated care (Figure 1).

**Messenger:** Different socio-cultural contexts will determine who is the most influential party to communicate the importance of collecting, managing, analysing and sharing insights from integrated care. It is important to be clear about who will be the most powerful and impactful messenger.

**Incentives:** Monetary incentives may help reinforce positive behaviour but understanding how integrated teams want to act collectively around the shared purpose of the best quality care for people and populations and rewarding that through integrated feedback and recognition is probably even more powerful.

**Norms:** Creating new integrated care professional norms and recognising them as highly valued, creates a cultural climate that reinforces the importance of sharing data collection and insight.

**Defaults:** People choose the path of least resistance and the one they are used to following. If integrated information requires too much effort, no matter how important they think it is to do it, they will prioritise what is easier.

**Salience:** Integrated care teams need to understand new information that is relevant to the delivery of coordinated care and case management. Designing data collection and information reporting with these needs in mind creates greater levels of buy-in from integrated care practitioners that the information will help deliver a constantly improving and innovating service. This same influence works for managers and payers.

**Priming and affect:** Kahneman [10] talks about system 1 and system 2 thinking where system 1 is fast and based on instinct and emotion. Integrated information systems need to be designed with the user in mind from the beginning with an understanding of what matters to them and people they are working with.

**Commitments:** Integrated care teams are usually highly motivated as individuals and collectively to solve the challenge of care fragmentation and resulting quality deficits. This commitment should be reflected in the design of integrated information systems providing teams with feedback that reinforces that commitment.

**Ego:** Integrated care teams, managers and payers want to feel that they are doing good for the populations they serve, seeing that reflected in information is a critical feedback loop.

## Conclusions

Where we have seen real successes in integrated information collection, management, analysis and reporting for integrated care, the starting point has always been understanding the needs of the users, be they the integrated care team (including the person in receipt of care) coordinating care, the service manager planning priorities, or the payer monitoring the impact of the integrated care on population health. Armed with this information, integrated care systems can design integrated information systems that:

1. Meet the needs of integrated care teams at the front line;
2. Create an environment that reinforces sharing of information for the good of the individual and community through salient feedback; and
3. Reward learning from the feedback of information creating a culture of collaborative continuous improvement.

My experience of working with integrated care systems at different stages of maturity [11] has demonstrated that the starting point has to be a more consistent approach to understanding the needs of the different users of integrated information in health and care. Taking a genuinely user-centred approach to design is new to most health systems despite years of talking about user-centricity. Behvioural insight driven design is become more common in social care internationally and there are encouraging signs that healthcare is starting to follow.
